# The Genome and Methylome of a Beetle with Complex Social Behavior, *Nicrophorus vespilloides* (Coleoptera: Silphidae)

**DOI:** 10.1093/gbe/evv194

**Published:** 2015-10-09

**Authors:** Christopher B. Cunningham, Lexiang Ji, R. Axel W. Wiberg, Jennifer Shelton, Elizabeth C. McKinney, Darren J. Parker, Richard B. Meagher, Kyle M. Benowitz, Eileen M. Roy-Zokan, Michael G. Ritchie, Susan J. Brown, Robert J. Schmitz, Allen J. Moore

**Affiliations:** ^1^Department of Genetics, University of Georgia; ^2^Institute of Bioinformatics, University of Georgia; ^3^Centre for Biological Diversity, School of Biology, University of St. Andrews, Fife, United Kingdom; ^4^Division of Biology & Bioinformatics Center & Arthropod Genomics Center, Kansas State University

**Keywords:** burying beetle, epigenetics, parental care, sociality

## Abstract

Testing for conserved and novel mechanisms underlying phenotypic evolution requires a diversity of genomes available for comparison spanning multiple independent lineages. For example, complex social behavior in insects has been investigated primarily with eusocial lineages, nearly all of which are Hymenoptera. If conserved genomic influences on sociality do exist, we need data from a wider range of taxa that also vary in their levels of sociality. Here, we present the assembled and annotated genome of the subsocial beetle *Nicrophorus vespilloides*, a species long used to investigate evolutionary questions of complex social behavior. We used this genome to address two questions. First, do aspects of life history, such as using a carcass to breed, predict overlap in gene models more strongly than phylogeny? We found that the overlap in gene models was similar between *N. vespilloides* and all other insect groups regardless of life history. Second, like other insects with highly developed social behavior but unlike other beetles, does *N. vespilloides* have DNA methylation? We found strong evidence for an active DNA methylation system. The distribution of methylation was similar to other insects with exons having the most methylated CpGs. Methylation status appears highly conserved; 85% of the methylated genes in *N. vespilloides* are also methylated in the hymentopteran *Nasonia vitripennis*. The addition of this genome adds a coleopteran resource to answer questions about the evolution and mechanistic basis of sociality and to address questions about the potential role of methylation in social behavior.

## Introduction

Understanding phenotypic evolution necessitates investigating both the ultimate and proximate influences on traits; however, these investigations require the appropriate tools. Social behavior is a particularly thorny phenotype to study because of its complexity, variation, and its multilevel integration across an organism (Boake et al. 2002). In addition, social behavior also often displays unusual evolutionary dynamics arising from the genetic influences on interactions required for sociality (McGlothlin et al. 2010). Although single genes can influence behavior (Fischman et al. 2011), social behavior is often multifaceted and can reflect a complex genetic architecture (Walling et al. 2008; Mikheyev and Linksvayer 2015) including influences from epigenetic mechanisms (Cardoso et al. 2015). Genomes in particular are useful resources for evolutionary questions of social behavior because they grant access to both broad scale and fine scale details and mechanisms (Richards 2015). For social behavior, although there are multiple Hymenopteran genomes available to investigate fine scale detail, we lack sufficiently distantly related species to address broader patterns. It is therefore important to develop genomic resources for organisms that are particularly useful phenotypic models of social behavior but where genomic information is lacking.

The genomes of several social insects are now available, including eusocial species such as honey bees (Elsik et al. 2014), stingless bees (Kapheim et al. 2015), several ant species (Gadau et al. 2012), primitively eusocial species including bumble bees (Sadd et al. 2015), a sweat bee (Kocher et al. 2013) and a euscocial termite (Terrapon et al. 2014). There is an assembled and annotated genome for the African social velvet spider (Sanggaard et al. 2014). Although enormous progress has been made in identifying genes associated with the behavioral division of labor and developmental shifts in social and other behavioral tasks in eusocial insects (Zayed and Robinson 2012; Rehan and Toth 2015), and the influence of epigenetic inheritance on developmental plasticity and behavior (Glastad et al. 2015; Yan et al. 2015), the generality of any mechanism underlying social interactions requires information from insects reflecting other levels of sociality and from other orders. Sociality occurs in nearly every insect order (Wilson 1971; Costa 2006), with eusociality representing an extreme on a social continuum. Outside Hymenoptera there are many subsocial species that have highly developed social behaviors, including parental care, but no division of labor (Costa 2006). To begin to address this gap, we assembled and annotated the genome of *Nicrophorus vespilloides*, a subsocial beetle that serves as a behavioral model species for many types of complex social interactions, including elaborate and advanced parental care with direct regurgitation of food to begging offspring (Walling et al*.* 2008<AQ7>), parent–offspring conflict (Kilner and Hinde 2012), sibling competition (Smiseth et al. 2007), and adult competition for resources (Hopwood et al. 2013). By sequencing, assembling, and annotating the genome of *N. vespilloides*, we were able to address two questions: First, is the gene complement of *N. vespilloides* more reflective of phylogeny or life history? Second, given methylation has been implicated in the success of eusocial species and facilitates plasticity, could this mechanism play a role in *N. vespilloides* social plasticity as well? *Tribolium castaneum*, the model beetle species, seems to lack DNA methylation (Zemach et al. 2010). This has led to the assumption that methylation may be unimportant in beetles generally. However, methylation has been suggested to regulate behavioral states in social insects (Glastad et al. 2015; Yan et al. 2015). *Nicrophorus vespilloides* is an unusual beetle in that it is highly social, with extensive interactions between parents and offspring, but males in the presence of females do not care for offspring or show the same levels of gene expression as caring parents (Parker et al. 2015). There is a rapid transition between behavioral states if the female parent is removed (Smiseth et al*.* 2005), with extensive changes in gene expression in the male (Parker et al. 2015). Given this, we sought to test for the presence of DNA methylation in *N. vespilloides*, which could provide a mechanism for this rapid behavioral transition.

**Fig. 1.—:**
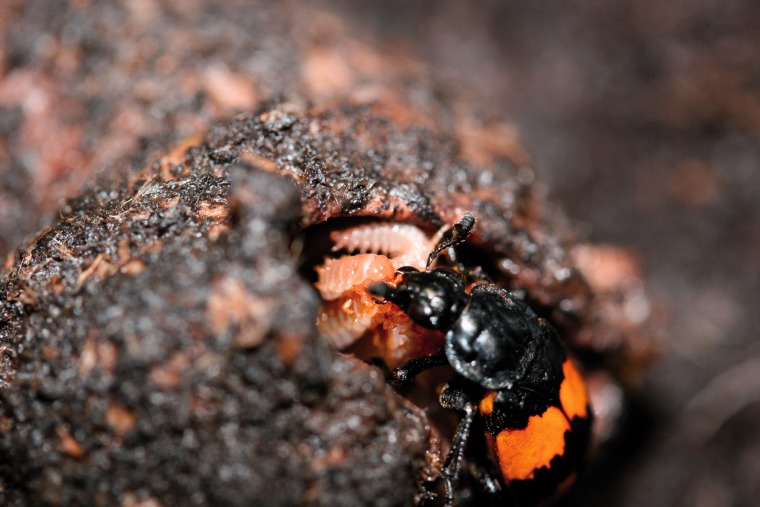
An adult female *N. vespilloides* regurgitating food into the mouth of her begging larvae on a prepared mouse carcass. Photograph by A. J. Moore.

Burying beetles (*Nicrophorus* spp.) are a group of about 85 species that are subsocial, showing a usual level of direct and indirect parental care of offspring (Eggert and Müller 1997; Scott 1998; Sikes and Venables 2013). Burying beetles use vertebrate carcasses as food for their offspring, and go well beyond simple forms of parental care with direct regurgitation of food by parents to begging offspring (Eggert and Müller 1997; Scott 1998; Walling et al. 2008; Trumbo 2012). There is also indirect parental care including depositing antimicrobial excretions to retard decomposition and microbial growth on the carcass used as food. Parents continuously maintain the carcass against microbial growth and interspecific competitors (e.g., fly larvae). The most extensively studied burying beetle, *N. vespilloides*, has proven an excellent model for investigating the ecology and evolution of social interactions between family members (Eggert and Müller 1997; Scott 1998; Trumbo 2012; fig. 1). Although parental care is essential, especially in the first 24 h of larval life (Eggert et al. 1998; Smiseth et al. 2003), care in this species can be uniparental, either male or female, or biparental. All forms of care are equivalently beneficial for offspring (Parker et al. 2015).

Here, we report a genome assembly and annotation of *N. vespilloides* and use this to investigate hypotheses regarding evolution associated with social behavior and the unusual life history of this beetle. Our assembly integrates high-throughput short reads, long reads, and a genome map providing sequence for greater than 90% of the predicted genome size. We annotated 13,526 protein-coding genes and compared these genes to social insects, another beetle, and a fly that uses vertebrate carcasses as food but lacks sociality. The rationale was to test whether social evolution, shared aspects of life history such as using carcasses for developing larvae, or shared evolutionary history is associated with similar molecular evolution. The overlap of shared number of orthologs was similar between *N. vespilloides* and all other insect groups regardless of the use of carcass for reproduction or highly developed social interactions. We then tested whether *N. vespilloides* has DNA methylation by looking for sequences coding for the enzymes responsible, DNA methyltransferases, and by using whole-genome bisulfite sequencing with the hypothesis that like *T. castaneum* (Zemach et al. 2010), *N. vespilloides* would lack DNA methylation. We confirmed the lack of methylation in *T. castaneum* but we did find evidence of DNA methylation in *N. vespilloides*. We found that the genes methylated in *N. vespilloides* showed considerable overlap with those methylated in a Hymenopteran, the jewel wasp, *Nasonia vitripennis*. Thus, the *N. vespilloides* genome adds the first coleopteran resource to investigators interested in the genomic and molecular signatures of social interactions, parent–offspring conflict, social tolerance, mate choice, and mate cooperation with an experimentally tractable and evolutionarily divergent model to use in comparative studies.

## Materials and Methods

### Animals Samples

All *N. vespilloides* used in this research were obtained from an outbred colony maintained at the University of Georgia under laboratory conditions for this species (see Cunningham et al. 2014 for a full description of conditions).

### Genome Size Estimation, Sequencing, Assembly, and Quality Control

We used flow cytometry with propidium iodide staining to estimate the genome size of *N. vespilloides* using *T. castaneum* as a standard. Nuclei from insect heads and whole insects, respectively, were prepared as described in Yu et al*.* (2015), stained as in Hare and Johnston (2011), and analyzed with a CyAn Flow Cytometer (Beckman Coulter, Brea, CA) at the UGA’s Center for Tropical and Emerging Global Diseases Flow Cytometry Core Facility. Data were processed with FlowJo software (Treestar, Inc., Ashland, OR).

Genomic DNA was extracted from a single larva derived from a single sibling–sibling mating using a sodium dodecyl sulfate-lysis buffer and a phenol–chloroform extraction. A 275-bp Illumina (San Diego, CA) TruSeq library was prepared and run on one lane of an Illumina HiSeq 2000 using a paired-end (2 × 100 bp) sequencing protocol at the HudsonAlpha Genome Sequencing Center (Huntsville, AL).

We used FastQC (v0.11.2; Babraham Institute; default settings) to create summary statistics and to identify possible adapter contamination of raw Illumina paired-end reads. No adapter contamination was reported, a result supported by analysis with CutAdapt (v1.2.1; Martin 2011), which only found evidence for adapters in less than 0.01% of the raw reads. Because sequencing library construction can generate inserts of genomic DNA that are less than twice the average read length, overlapping paired-end reads were first merged using FLASH (v1.2.4; Magoc and Salzberg 2011; default settings, insert size: 278 bp with SD of 53 bp [estimate from Platanus scaffolding step]). Quality control was performed with PrinSeq (Schmieder and Edwards 2011b). Reads were required to have a mean overall Phred quality score of ≥25, read ends were trimmed to >20 Phred quality score, a minimum length of 90 bp and a maximum length of 99 bp were allowed, and reads were allowed only one unidentified (N) nucleotide per read.

To obtain Pacific Bioscience (PacBio; Menlo Park, CA) continuous long reads (CLRs), we extracted genomic DNA using the same phenol–chloroform extraction as used to extract gDNA for the Illumina sequencing from a brother/sister pair of adult beetles that had been inbred for six generations. The University of Maryland Institute for Genomic Sciences prepared a 14.4-kb-long insert PacBio library. This library was sequenced with 22 PacBio’s RS II P5-C4 Single Molecule, Real Time (SMRT) cells to generate CLRs to scaffold the assembly to increase long-range connectivity of the assembly. PacBio reads greater than 6,300 bp (36.4× coverage) were error corrected with the PBcR pipeline (Koren et al. 2012) using 49× coverage of the quality-controlled Illumina reads with default settings, which after error correction and assembly produced an estimated 20.9× coverage of CLRs.

To increase the long-range scaffolding (i.e., superscaffold) of our draft genome, we generated a BioNano Genomics (San Diego, CA) genome map. High molecular weight (HMW) genomic DNA was extracted from a single pupa as previously described (Shelton et al. 2015). HMW gDNA was nicked with a nicking restriction digest by *Bsp*QI and *Bbv*CI restriction enzymes that had been converted to nickases (New England Biolabs, Ipswich, MA). Restriction sites were labeled with fluorescent nucleotides and imaged on the Irys system (BioNano Genomics) according to the manufacturer’s instructions.

All Illumina reads passing quality control were used as input for the Platanus assembler (v1.2.1; Kajitani et al. 2014). First, reads were assembled into contigs using the assemble protocol (nondefault settings: -s 3 -u 0.2 -d 0.3 -m 128). Next, contigs were scaffolded using the scaffold protocol (nondefault settings: -u 0.2). This step was iterated a total of five times using the same settings to extend the scaffold as much as possible with the Illumina reads. Gaps in the assembly were filled using the gap_close protocol with default settings. This step was iterated twice. Only contigs/scaffolds 1 kb or greater in length were used for further analysis and assembly.

PacBio reads were used to gap fill and scaffold the Platanus assembly with PBJelly2 (v14.9.9; English et al. 2012) using default settings and the error-corrected PacBio CLRs.

A genome map created from BioNano Genomic single molecule maps was used to superscaffold the Platanus/PBJelly assembly (Shelton et al. 2015). The BioNano Genomics genome map provides a means to “superscaffold” an assembly by using HMW DNA that has been fluorescently labeled at specific sequence recognition sites that is then compared with in silico maps of the assembly to link scaffolds over very large genomic distances. It also provides an independent validation of a genome assembly. Briefly, the images were assembled into a consensus map based on the labeling pattern of each molecule imaged. These in silico maps, with a cumulative length of 133.7 Mb, were compared with the predicted labeling pattern of the Platanus/PBJelly that passed a quality filter (length > 150 kb and number of labels ≥ 8) to further scaffold and orient the Platanus/PBJelly assembly.

DeconSeq (v0.4.2; Schmieder and Edwards 2011a) was used to assess our draft assembly for possible contamination. Besides the 1,126 bacterial species included in the distribution, we also updated the human genome sequence (h37) and added the genomes of *Caenorhabditis elegans*, *Ralstonia pickettii*, and *Yarrowia lipolytica*. *Caenorhabditis elegans* was included because it is the closest genome available to the nematode symbiont of *N. vespilloides*, *Rhabditis stammeri* (Richter 1993). *Ralstonia pickettii* and *Y. lipolytica* were included because they were two species that showed up when the RNA-Seq experiment was assessed for possible contamination (Parker et al. 2015). *Tribolium castaneum* was used as a retention database. Only one contig was flagged and removed during our contamination search; belonging to *Morganella morganii*, a common bacterium found in vertebrate intestinal tracts.

Genome assembly quality and completeness were assessed with multiple benchmark data sets. First, the CEGMA analysis pipeline (v2.4.010312; Parra et al. 2009) was used to assess the completeness of 248 ultra conserved eukaryotic genes within our assembly. Next, we used the *T. castaneum* set of Benchmarking sets of Universal Single-Copy Orthologs (BUSCOs; 2,787 genes) to further assess the assembly completeness (Waterhouse et al. 2013). We also mapped the RNA-Seq reads back to the assembly to estimate coverage of the transcriptome of our assembly using the TopHat (v2.0.13) pipeline with Bowtie2 (v2.2.3) as the read aligner.

### Genome Annotation

To begin genome annotation, we first generated a de novo library of repeats using Repeat-Modeler (v1.0.8; Smit and Hubley 2014) that integrates three separate repeat finder programs; RECON (v1.08; Bao and Eddy 2002), RepeatScout (v1.05; Price et al. 2005), and TRF (v4.07b; Benson 1999) with default parameters. Because some gene fragments, especially low-complexity motifs, might be captured in the repeat analysis, we used BLASTx to remove any matches to *T. castaneum* proteins in the UniProtKB database (Wang et al. 2008; Jiang 2014). The repeat analysis of the *T. castaneum* genome was carried out with RepeatMasker (v4.0.5; Smit et al. 2015) using default settings.

We annotated putative protein-coding genes using the Maker2 annotation pipeline (v2.31.7; Holt and Yandell 2011) using an iterative process. After masking putative repeats within a genome, this pipeline generates gene models, including 5′- and 3′-untranslated regions (UTRs), by integrating ab initio gene predictions with aligned transcript and protein evidence. First, we mapped and assembled transcripts using the RNA-Seq data from an experiment of *N. vespilloides* in multiple behavioral states over a breeding cycle (mated, caring, and postcaring; see Parker et al. 2015 for full details) using the Bowtie (v2.2.3)/TopHat (v2.0.13)/Cufflinks (v2.2.1) pipeline (Langmead et al. 2009; Trapnell et al. 2010; Kim et al. 2013). To begin the annotation process, we annotated the genome exclusively with the *N. vespilloides* Cufflinks-assembled transcripts and the proteomes from five insects (*T. castaneum, Na. vitripennis, Apis mellifera, Musca domestica,* and *Drosophila melanogaster*; downloaded from UniProtKB, including all isoforms for comprehensive coverage) using default parameters, except for est2genome=1, protein2genome=1. After this first iteration of annotation (and every subsequent iteration), three scaffolds were inspected to visually check for annotation biases (Hoff and Stanke 2015) using the Apollo genome browser (Lewis et al. 2002). The next iteration used the same input data and parameters, except changes to split_hit=2000, correct_est_fusion=1, which corrected for the smaller intron size observed and the propensity of MAKER to fuse gene models that likely should be separate as inferred by visual inspection of BLAST evidence. For the next iteration, three ab initio gene predictors were included in the annotation process: Augustus (v2.5.5; Stanke et al. 2006), GeneMark-ES (v4.21; Lomsadze et al. 2005), and SNAP (v2010-7-28; Korf 2004; using est2genome=0, protein2genome=0). With AUGUSTUS, we used the “tribolium” gene set provided with its distribution to guide gene predictions. GeneMark was trained on the draft assembly of the *N. vespilloides* genome sequence using its automated training routine. SNAP was trained using the MAKER2 gene models produced during the first round of annotation. All gene predictors were run with default parameter values. The annotation was iterated twice with the gene predictors, updating the SNAP HMMs between the two iterations. Transfer RNAs were identified using tRNAscan-SE (v1.23; Lowe and Eddy 1997) within the Maker2 pipeline during the last iteration. Other noncoding RNA (ribosomal RNA, microRNA, small nuclear RNA, and small nucleolar RNA) were predicted and annotated with INFERNAL (v1.1.1; Nawrocki and Eddy 2013) using the complete Rfam database (v12.0; Nawrocki et al. 2014; supplementary table S4, Supplementary Material online).

### Functional Annotation of Predicted Protein-Coding Genes

To gain insight into the putative function of each gene model, we annotated our gene models with three pipelines. First, we used BLASTp (v2.2.26; Altschul et al. 1997) to find the best hit against the entire UniProtKB database (vJan15; *E* value: 10e-5). Next, we used InterProScan (v5.8-49.0; Hunter et al. 2009) to find the known protein domains within every gene model from the TIGRFAM, ProDom, SMART, TMHMM, Phobius, PANTHER, PrositeProfiles, SignalP-EUK, SuperFamily, PRINTS, Gene3d, PIRSF, Pfam, and Coils databases. We also used InterProScan5 to assign gene ontology (GO) terms to further characterize each protein. KEGG pathway analysis was also performed using the KEGG Automatic Annotation Server (KAAS; Moriya et al. 2007) using the single-directional best hit method to assign orthology with default parameters and the default Eukaryote gene sets plus all available arthropod gene sets.

### Ortholog Comparison

To compare the orthology of our gene models to other insects, we analyzed our final MAKER2 proteome using OrthoMCL (v2.0.9; Li et al. 2003) against five other insect proteomes. We compared with *T. castaneum* and *D. melanogaster* as model inset genomes, *Na. vitripennis* and *A. mellifera* as other insects that share a social life history, and *M. domestica* as an insect that shares the use of carrion for reproduction and food for developing young. If a gene was represented by more than one isoform in its respective official gene set (OGS), the longest isoform was chosen for this analysis. We used BLASTp (*E* value: 1e-5) to characterize the homology among all proteins. The output from this analysis was used by OrthoMCL to cluster proteins into orthologous groupings. Results are presented as Venn diagrams generated using the University of Ghent Bioinformatics Evolutionary Genomics’ Venn Diagram webtool (http://bioinformatics.psb.ugent.be/webtools/Venn/, last accessed April 16, 2015).

### Gene Family Expansion/Contraction Analysis

To investigate possible expansion and contraction of shared gene families of the six insects that we used in the OrthoMCL analysis, we used CAFÉ (v3.1; default settings; Han et al. 2013) with phylogenetic relationships from Trautwein et al*.* (2012) and divergence times from TimeTree (Hedges et al. 2006). Only gene families with at least one representative from *N. vespilloides* were considered as gene family contractions.

Enrichment of GO terms among the expanded gene family members was performed using argiGO’s web-based Singular Enrichment Analysis (Du et al. 2010) of customized annotations by comparing the GO terms associated with methylated gene from the InterProScan results to all GO terms associated with all genes from InterProScan. Specifically, a hypergeometric test with a Benjamini–Hochberg false discovery rate (FDR) correction at a familywise error rate of 0.05 was applied after GO terms were converted into generic GO slim terms. All other parameters were set at default values.

### Selection Analysis

To assess the rates of molecular evolution within the *N. vespilloides* genome, we used PAML (Yang and Bielawski 2000; Yang 2007) to calculate d*N*, d*S*, and their ratio (ω) and compare these metrics to the beetles *T. castaneum* and *Dendroctonus ponderosae*. We identified a set of 1:1 orthologs between *N. vespilloides, D. ponderosae* and *T. castaneum* using a combination of the BLAST (Basic Local Alignment Search Tool) (Altschul et al. 1997; Camacho et al. 2009), orthAgogue (Ekseth et al. 2014), and mcl (Enright et al. 2002; van Dongen 2008) as well as part of the OrthoMCL (Li et al. 2003) pipeline. In total, 5,584 orthologs between all three species were recovered. Amino acid sequences for each were aligned in PRANK (v100802; Löytynoja and Goldman 2005). Codeml in the PAML package was used to test different models of molecular evolution for each gene. Our interest is in determining which genes show evidence of a differential rate of evolution within *N. vespilloides*. We therefore tested a basic model (model = 0, NSsites = 0, fix_omega = 0) that assumes a single *ω* across all the entire phylogeny against a branch model (model = 2, NSsites = 0, fix_omega = 0), which assumes one ω for the *N. vespilloides* branch and another ω for the branches to *T. castaneum* and *D. ponderosae*. These models are compared, for each gene, with a likelihood ratio test with 1 degree of freedom. We then adjusted the significance threshold for a gene to show statistically significant different rates of sequence evolution using a Benjamini–Hochberg FDR correction at *q* of 0.05 (Benjamini and Hochberg 1995). Finally, any estimates of d*S,* d*N* or ω >10 were discarded. These species are phylogenetically distant (240 Ma) and this increases the likelihood signals of molecular evolution will be lost due to saturation of d*S*.

### DNA Methylation Analysis

As the first step to characterize if DNA methylation existed within *N. vespilloides*, we use BLASTp (Altschul et al. 1997) to identify putative DNA methyltransferases. We search our genome with known members of Dnmt families of both vertebrate (*Mus musculus*; 1, 2, 3a, 3b, 3 l) and invertebrate (*T. castaneum, A. mellifera, D. melanogaster*; 1, 2, and 3). After three putative loci were found (one member per Dnmt family), we further characterized the possible functional relationship of the proteins by clustering them with the BLAST query proteins and several more invertebrate species (*Zootermopsis nevadensis* and *Camponotus floridanus*) using ClustalW followed by a neighbor-joining tree with 10,000 bootstraps in CLC Sequence Viewer (v7.5; http://www.clcbio.com) with default settings.

To address whether DNA methylation is present in *N. vespilloides*, we performed methylC-Seq (Lister et al. 2008), whole-genome sequencing of bisulfite-treated genomic DNA, on three biological replicates of whole larvae to create single base resolution of DNA methylation, if present. DNA was extracted from three whole *N. vespilloides* larvae, respectively, using the same protocol as for the Illumina and PacBio sequencing (see above). Due to previous reports that *T. castaneum* contains no DNA cytosine methylation (Zemach et al*.* 2010), samples from this species were used as a negative control and DNA was extracted from three biological replicates that each contained at least 15 pooled whole larvae using the same protocol as for *N. vespilloides*. methylC-Seq libraries were prepared according to the protocol of Urich et al*.* (2015). Deep sequencing was performed using an Illumina NextSeq500 Instrument at the University of Georgia Genomics Facility (supplementary table S5, Supplementary Material online).

Raw fastq files were trimmed for adapters CutAdapt (v1.3) and preprocessed to remove low-quality reads. We aligned quality-controlled reads to the *N. vespilloides* v1.0 and *T. castaneum* v3.0 reference genomes using the method as described in Schmitz et al*.* (2013). The *T. castaneum* genome and OGS gff (v3.0) were obtained from BeetleBase.org. Lambda sequence (which is fully unmethylated) was used as a control to calculate the efficiency of the sodium bisulfite reaction and the associated nonconversion rate of unmodified cytosines, which ranged from 0.10% to 0.11% (supplementary table S5, Supplementary Material online). Only cytosine sites with a minimum coverage of three reads were used for subsequent analysis. A binomial test coupled with a Benjamini–Hochberg FDR correction at a familywise error rate of 5% was used to determine the methylation status of every cytosine (Benjamini and Hochberg 1995). Weighted methylation levels were calculated as previously described (Schultz et al. 2012).

We next characterized the distribution of methylated cytosines across the *N. vespilloides* genome and gene models. Methylated cytosines and their flanking two bases were extracted out for sequence conservation analysis using the program WebLogo 3.3 (Crooks et al. 2004). To perform the symmetry analysis, both strands of each CpG dinucleotide were required to have a minimum coverage of at least three reads and at least one of the CpG sites was identified as methylated. Upstream regions were defined as 1 kb upstream starting from the translational start site or the transcriptional start sire if a 5′-UTR was annotated. The program bedtools was used to determine the distribution of methylated CpG sites (Quinlan and Hall 2010). We used a two-step process to identify “methylated” and “unmethylated” genes. First, the probability of a methylated CpG site occurring within a gene was determined by totaling all methylated CpG sites within all genes and dividing this value by the total mapped CpG sites within all genes. Second, the methylated CpG sites and mapped CpG sites of each gene were used to determine that gene’s methylation status using a binomial test. These results were then corrected for multiple testing using a Benjamini–Hochberg FDR correction at 5%. Only genes with at least five mapped CpG sites were reported. *Nicrophorus vespilloides* replicate 1 was used to compute the exact values and percentages, but all replicates were qualitatively similar (supplementary table S6, Supplementary Material online).

To compare with previous documented signatures of methylation in insects, we calculated CpG_O/E_ ratios for each gene following the method described in Elango et al. (2009), a ratio of the observed level of methylation in genes over expected levels given the GC content of the genes analyzed. Thus, CpG_O/E_ is a normalized measure of depletion of CpG dinucleotides. Following Elango et al. (2009), the CpG_O/E_ for each gene was calculated as

$$\text{C}\mathrm{pG}{\;}_{\text{O}/\text{E}}=\;\frac{{\text{P}}_{\text{CpG}}}{{\text{P}}_{C}*{\text{P}}_{G}},$$

where P_CpG_ is the frequency of CpG dinucleotides, P_C_ is the frequency of cytosine, and P_G_ is the frequency of guanine estimated from each gene

Finally, we compared the genes that were methylated in *N. vespilloides* to another insect with a recently characterized active methylation system, *Na. vitripennis* (Wang et al. 2013). For direct comparison, we generated the *Na. vitripennis* results with their previously published data. We downloaded raw reads and mapped them to the published *Na. vitripennis* v1.0 reference genome and OGS v1.2. “Methylated” genes were established with the same protocol as we describe above for *N. vespilloides* to ensure that the comparison was appropriate. We only included single-copy ortholog that existed in both *N. vespilloides* and *Na. vitripennis* genomes in the comparison of the overlap between methylated gene sets.

## Results

### Genome Sequencing and Assembly

We assembled the genome of *N. vespilloides* by integrating evidence from Illumina short reads, Pacific Bioscience (PacBio) CLRs, and a BioNano Genomics genome map (supplementary table S1, Supplementary Material online). We assembled 195.3 Mb of the *N. vespilloides* genome, which is 95.7% of its predicted size (supplementary fig. S1, Supplementary Material online). The draft genome is contained within 5,858 contigs with an N50 of 102.1 kb and further into 4,664 scaffolds with an N50 of 122.4 kb (Longest scaffold: 1.80 Mb; table 1). The Illumina and PacBio data produced as assembly with a scaffold N50 of 115.4 kb and a longest scaffold of 989 kb. With the addition of the BioNano Genomics genome map, these metrics were increased to 122.4 kb and 1.795 Mb, respectively. The GC content is 32%, consistent with two other beetle genomes, *T. castaneum* at 33% (Tribolium Genome Sequencing Consortium 2008) and *D. ponderosae* at 36% (Keeling et al. 2013).

**Table 1:** Summary Statistics of *Nicrophorus vespilloides* Draft Genome Assembly

Total assembled length (bp)	195,308,655
Contigs (*n*)	5,858
Contig N50 (bp)	102,139
Largest contig (bp)	944,646
Scaffolds (*n*)	4,664
Scaffold N50 (bp)	122,407
Largest scaffold (bp)	1,795,199
% GC content	31.85
Predicted gene models (# of loci)	13,526
CEGMA pipeline analysis (% complete/partial)	99.6/100
Analysis of *N. vespilloides* with *Tribolium castaneum* BUSCO gene set	
Mean % sequence indenity shared	65.95
Mean % of *T. castaneum* gene length found	90
% of *T. castaneum* BUSCO genes found as single-copy orthologs	96.8
BioNano Genomics genome map alignment to in silico maps (%)	31

We assessed how well the protein-coding portion of the genome was assembled using the CEGMA and BUSCO pipelines. Our genome contained 247 complete orthologs (99.6%) and 248 partial orthologs (100%) of the CEGMA proteins. Of the 2,827 *T. castaneum* BUSCO proteins, our genome contained 2,737 (96.8%) as single-copy orthologs and 86 (3.1%) as multicopy orthologs. We also mapped the RNA-Seq data used for annotation back to the genome to assess transcriptome coverage. There was an 89.7% mapping rate.

### Genome Annotation

We used Maker2 to annotate the protein-coding portion of the genome by integrating ab initio, protein homology, and species-specific RNA-Seq evidence into consensus gene models. We obtained 13,526 predicted gene models. The gene models had an average protein length of 466.7 amino acids and 6.3 exons. Maker2 also predicted 5′-UTRs for 5,813 genes (mean: 512 bp) and 3′-UTRs for 4,549 genes (mean: 980 bp).

**Fig. 2.—:**
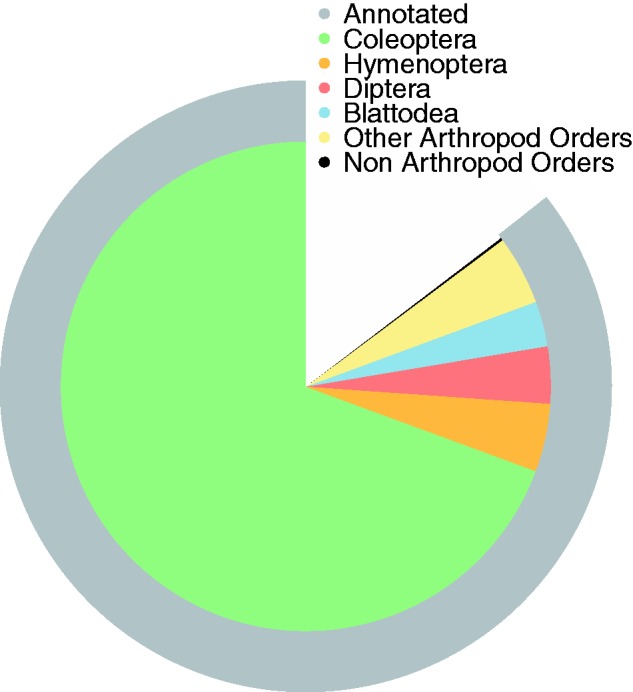
A two-ring pie chart showing results of annotation with BLAST against the complete UniProtKB database. First outer ring (gray) shows the proportion of gene models that could be annotated. Second ring (multicolored) shows the proportion of best BLAST hits of the annotations by order for all species with five or more best hits (97.8%). The best BLAST hits were overwhelmingly from other beetles and other Arthropods.

We were able to functionally annotate 11,585 gene models (85.6%) against UniProtKB with BLASTp. Restricted to species that had five or more best matches against *N. vespilloides* (encompassing 97.8% of the annotated gene models), the annotated gene models overwhelmingly returned the strongest similarity to other Coleoptera (fig. 2 and supplementary table S2, Supplementary Material online; top three species—*T. castaneum*: 6,969, *D. ponderosae*: 1,368, *Anoplophora glabripennis*: 743; Coleopteran total: 9,210 [79.5%]). Arthropods were the strongest similarity matches for 11,305 (99.7%) gene models (fig. 2). We were also able to identify at least one protein domain in 86.1% of the genes using InterProScan5. Searches against the Pfam database found 9,467 domains from 3,932 unique families. We were also able to assign at least one GO term to 7,492 genes (55.4%). Additionally, we were able to associate KEGG orthology terms with 44.8% of the genes.

Our de novo repeat analysis found that 12.85% of the draft genome is composed of repetitive elements. The top three classifications of repeats were unclassified repetitive elements (6.13%), DNA elements (3.35%), and simple repeats (2.24%). The overall repeat content is lower than that reported for beetles *T. castaneum* (Tribolium Genome Sequencing Consortium 2008) and *D. ponderosae* (Keeling et al. 2013), but higher than the honey bee (Elsik et al. 2014) and the red harvester ant (Smith et al. 2012), all of which have genomes that are of comparable size to *N. vespilloides*. Additionally, when we provided our repeat library to RepeatMasker to mask the *T. castaneum* genome only 1.65% was masked, an outcome consistent when the repeat library of *D. ponderosae* was used for the same task (0.15% of *T. castaneum* masked; Keeling et al. 2013; supplementary table S3, Supplementary Material online).

### Orthology of Gene Models

We used OrthoMCL, which clusters proteins based on a reciprocal best BLAST hit strategy, to assign orthology of the *N. vespilloides* proteome against five other insect proteomes chosen either because they are genomic models (*T. castaneum* and *D. melanogaster*) or because they share a social life history (*A. mellifera* and *Na. vitripennis*) or the use of carcasses to breed and as food for offspring (*M. domestica*). Thus, these are simple and limited comparisons but they serve as a first enquiry into the forces that might shape genome evolution.

**Fig. 3.—:**
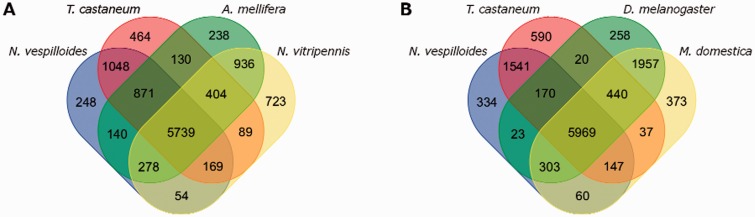
Figure shows the results of the OrthoMCL analysis that clustered the proteomes of *N. vespilloides*, *T. castaneum*, *A. mellifera*, *Na. vitripennis*, *D. melanogaster*, and *M. domestica* into orthologous groupings. (*A*) A Venn diagram showing the overlap in the orthologous groupings of the two beetles (*T. castaneum* and *N. vespilloides*) and the two Hymenoptera (*A. mellifera* and *Na. vitripennis*). (*B*) A Venn diagram showing the overlap in orthologous groupings of the two beetles (*T. castaneum* and *N. vespilloides*) and the two Diptera (*D. melanogaster* and *M. domestica*).

Our analysis produced 11,929 orthologous groupings with representatives from at least two different lineages. There were 4,928 orthologs groupings that contained at least one protein from each species. Of these, 3,734 groupings were single-copy orthologs among the six insects. There were 153 groupings containing 532 proteins that had proteins from *N. vespilloides* only. The beetles, *N. vespilloides* and *T. castaneum*, were represented in 7,827 groupings and 716 groupings were exclusive to beetles (650 were single-copy ortholog groupings). We then made two specific comparisons of the proteomes of *N. vespilloides*, *T. castaneum*, *A. mellifera*, *Na. vitripennis*, *D. melanogaster,* and *M. domestica* (fig. 3). *Nicrophorus vespilloides* shared 6,465 orthologous groupings with *D. melanogaster*, 6,479 with *M. domestica*, 7,028 with *A. mellifera*, and 6,240 with *Na. vitripennis*. We used a *z*-test to test whether the proportion of shared orthologous groupings was different between our two comparisons (*A. mellifera* vs. *Na. vitripennis* and *D. melanogaster* vs. *M. domestica*). *Nicrophorus vespilloides* shared more orthologous groupings with *A. mellifera* than with *Na. vitripennis* (*z* = 9.539, *P* < 0.001); however, *N. vespilloides* did not share more orthologous groupings exclusively with *A. mellifera* than *T. castaneum* (140 vs. 130, respectively; *z* = 0.613, *P* = 0.729). *Nicrophorus vespilloides* did not share more orthologous groupings with *M. domestica* than with *D. melanogaster* (*z* = −1.427, *P* = 0.156). However, *N. vespilloides* did share more orthologous groupings exclusively with *M. domestica* than *T. castaneum* (60 vs. 37, respectively; *z* = 2.341, *P* = 0.022).

### Gene Family Expansion and Contraction

To investigate whether there had been any gene family expansions or contractions in *N. vespilloides*, we analyzed the results of the OrthoMCL analysis with CAFÉ. There were 269 orthology groupings (or gene families) that showed significant expansion or contraction between the six insect species compared at *P* < 0.0001. Of these groupings 12 showed significant differences within the *N. vespilloides* lineage. There were eight expansions and four contractions (supplementary file S1, Supplementary Material online). The expansions were mostly families of uncharacterized proteins (7/8), whereas the last family was a chymotrypsin protease. There was not an enrichment of any GO term from the expanded gene families. The contracted families had highest similarity to an esterase, a transposase, a cytochrome P450, and an uncharacterized protein in *T. castaneum*. Some of these are also differentially expressed during caring (Parker et al. 2015).

### Selection Analysis

Signatures of selection on the protein-coding genes of *N. vespilloides* were investigated by comparing the d*N/*d*S* (*ω*) ratio to *T. castaneum* and *D. ponderosae* for the 5,584 one-to-one orthologs we detected between these lineages. Twenty-five genes showed signs of differential divergent selection in the *N. vespilloides* lineage after our filtering criteria were applied (see supplementary fig. S2, Supplementary Material online; BH FDR = 0.05 and removal of genes showing d*N*, d*S*, or ω >10). Two genes show evidence of positive selection *ω* > 1: Ephrin-B2 (*efn-b2*; *ω* = 1.45) and NK Homeobox (HOX) 7 (*nk7*; *ω* = 2.16). *efn-b2* also has a *ω* > 1 in the other lineages (*ω* = 1.5), whereas *nk7* shows evidence of strong conservation in the *T. castaneum* and *D. ponderosae* lineages. The median estimates of d*S*, d*N* and *ω* were higher in the *N. vespilloides* lineage (*N. vespilloides*: 0.0489, *T. castaneum:* 0.0487, and *D. ponderosae*: 0.0487), although not statistically significantly different.

### DNA Methylation

We used two approaches to investigate whether the *N. vespilloides* genome has active DNA methylation. First, we looked for the enzymes responsible for methylation in animals (Dnmt1, Dnmt2, and Dnmt3) to determine whether the machinery was present for the establishment and maintenance of DNA methylation. Second, we generated single-base resolution maps of DNA methylation using whole-genome bisulfite sequencing.

**Fig. 4.—:**
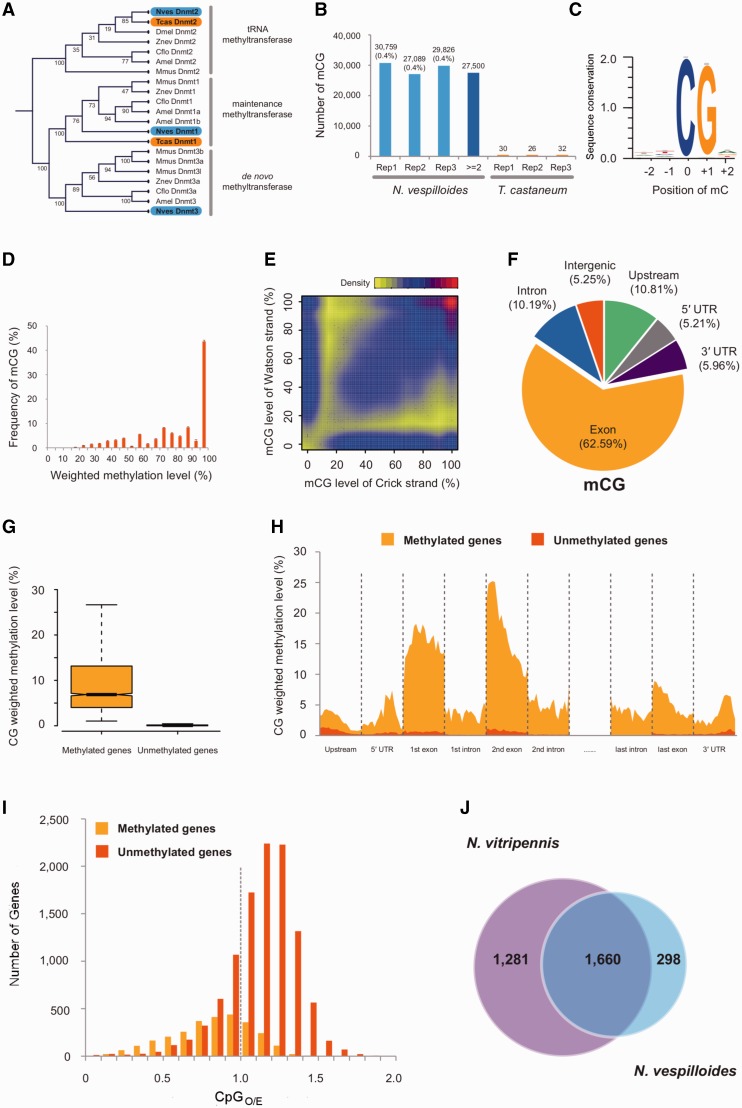
Summary of DNA methylation analyses. (*A*) A cladogram showing the relationship of the Dnmt’s across several insects and a mammal. Nves, *N. vespilloides*; Tcas, *T. castaneum*; Dmel, *D. melanogaster*; Cflo, *C. floridanus*; Amel, *A. mellifera*; Mmus, *M. musculus*; Znev, *Zootermopsis nevadensis*. (*B*) Number of methylated cytosines in each of the three replicates of *N. vespilloides* and *T. castaneum*. (*C*) A sequence logo of the overwhelming occurrence of methylation in CpG dinucleotide by showing the nucleotide proportions of the two nucleotides both upstream and downstream of the methylated cytosines. (*D*) A histogram of CpGs that are considered methylated versus the proportion of reads that supported their methylation status (weighted methylation level). (*E*) A density plot showing the very high symmetry of methylated CpG sites on opposing strands of DNA. (*F*) A pie chart showing the distribution of methylated CpGs across gene elements. (*G*) A standard box plot of the proportion of reads that supported methylation status (weighted methylation level) with genes grouped by whether they were methylated or not. (*H*) Diagram showing the proportion of reads that supported methylation (weighted methylation level) of methylated and unmethylated genes across each region of a gene model summarized as 20 bins within a region. (*I*) Histograms of methylated and unmethylated genes versus the CpG observed/expected ratio across a gene body. (*J*) Venn diagram illustrating the overlap of methylated genes that had 1:1 orthology between the burying beetle *N. vespilloides* and the jewel wasp *Na. vitripennis*.

Single copies of all three DNA methyltransferases were in the *N. vespilloides* genome; *T. castaneum* contains only Dnmt1 and Dnmt2 (Kim et al. 2010). The methyltransferases clustered with their putative orthologs (fig. 4*A*). Next, using MethylC-Seq we found direct evidence for DNA cytosine methylation in *N. vespilloides* (mean = 29,224 methylated cytosines) and no evidence for DNA methylation in *T. castaneum* (mean = 29 methylated cytosines), supporting previous reports on the latter (fig. 4*B*; Zemach et al. 2010). Methylation (5′-methlycytosine) in *N. vespilloides* was found within a CpG context exclusively (fig. 4*C*). A small proportion (1.87%) of CpH (H = A, T, or C) was found during the first analysis; however, further analysis of the originally identified CpH methylated sites revealed that greater than 98% of them were artifacts of segregating single nucleotide polymorphisms. Therefore, only strong evidence was found for CpG methylation in the genome. Methylated cytosines in *N. vespilloides* exhibited the typical insect pattern where most mapped reads at a given locus provided support for methylation or not (fig. 4*D*) and a high level of symmetrical methylation on opposing DNA strands (fig. 4*E*). The genome-wide pattern of DNA methylation observed for *N. vespilloides* is also similar to other insects. Most prominently, the majority of methylation was found within genic regions (94.75% of the observed methylation) and further within the exons (62.55 ± 0.26% of the observed methylation) and much lower levels were found in introns (10.29 ± 0.12% of the observed methylation; fig. 4*F*). All three biological replicates are quantitatively similar in their distribution of methylated CpGs over gene elements (supplementary table S6, Supplementary Material online). We grouped *N. vespilloides* genes as methylated or unmethylated by comparing the level of methylation of an individual gene with the average level of gene methylation found across all genes. We found 2,782 genes that were methylated significantly higher than the null expectation (fig. 4*G* and supplementary file S2, Supplementary Material online). Following this, we performed a GO term enrichment analysis on the GO terms associated with the methylated gene set. We found that nucleic acid binding (GO:0003676), translation factor activity/nucleic acid binding (GO:0008135), and RNA binding (GO:0003723) were significantly enriched molecular function GO terms. Cellular macromolecule metabolic process (GO:0044260), cellular protein metabolic process (GO:0044267), and macromolecule biosynthesis process (GO:043170) were the three most enriched biological process GO terms (see also supplementary table S7, Supplementary Material online). At the level of individual genes, methylation was highest in the exons (fig. 4*H*). Methylation was also observed in the 5′- and 3′-UTRs, with the typical steep decrease in methylation observed at the translational start site. We also observed methylation in the “promoter” region 1 kb upstream from the first annotated gene element. Methylation peaks beginning at the second exon, although this is not a robust trend as methylation levels decrease to the same level of the first exon by the end of the second exon. Transposable elements were methylated to the same level as genomic intergenic background levels (3% vs. 5%, respectively).

Comparing patterns of methylation to other insects, we found that as expected methylated genes had lower CpG_O/E_ values compared with nonmethylated genes (fig. 4*I*). The mean of methylated genes was 0.82, whereas that for nonmethylated genes was 1.13. We further assessed how many of methylated genes overlapped in a Hymenopteran and the burying beetle. We found that there were 4,633 methylated genes in the jewel wasp *Na. vitripennis* and 2,782 *in N. vespilloides*. Of the 1,958 single-copy orthologs that were methylated in *N. vespilloides*, 85% overlapped with methylated genes in the jewel wasp (fig. 4*J*).

## Discussion

The ability to detect conserved and novel molecular mechanisms that influence social behavior requires genomic resources from species across different lineages that vary in their level of sociality. Here, we report the draft genome of *N. vespilloides*, a subsocial beetle from the Silphidae. In assessing the genetic changes associated with the evolution of social behavior in insects, the *N. vespilloides* genome provides a useful line of independent evolution, offering data from outside the Hymenoptera, which diverged from Coleoptera approximately 350 Ma (Wiegmann et al. 2009) and at a level between solitary and eusocial. *Nicrophorus vespilloides* has sophisticated and complex parental care (Eggert and Müller 1997; Scott 1998; Trumbo 2012). The highly developed social interactions between parents and offspring place this beetle at the level of “subsocial” on the evolutionary spectrum of social species (Wilson 1971; Costa 2006).

We successfully assembled the *N. vespilloides* genome using Illumina short reads, PacBio CLRs, and a BioNano Genomics genome map. Our assembly quality compares favorably with other recently published insect genomes; especially considering our organism is outbred (Richards and Murali 2015). We found that our genome is similar to other recently sequenced insect genomes, with a comparable number of genes and percentage of genes having a functional annotation (Kim et al. 2010; Wurm et al. 2012; Keeling et al. 2013; Oxley et al. 2014; Wang et al. 2014). Our orthology analysis showed that *N. vespilloides* was as similar to social Hymenoptera or to a Dipteran as it is to the asocial beetle *T. castaneum* with respect to the number of shared gene families. This is in contrast to the finding of expanded repertoire of immune genes and chemoreceptor genes in *M. domestica* compared with *D. melanogaster* (Scott et al. 2014).

Very few of the *N. vespilloides* genes we examined showed evidence of differential rates of sequence evolution compared with the *T. castaneum* and *D. ponderosae* lineages. Among the genes that did show differential d*N*/d*S* ratios, the majority showed low d*N*/d*S* values consistent with evolutionary conserved amino acid sequence (Yang and Bielawski 2000). We found only two genes with evidence of d*N*/d*S* >1, consistent with positive diversifying selection. NK HOX 7 had an elevated *ω* in the *N. vespilloides* lineage but is highly conserved in the other lineages. Ephrin-B2 had an elevated *ω* in all lineages but it is slightly lower in the *N. vespilloides* lineage. Both of these genes are involved in developmental patterning (Dönitz et al. 2015; Dos Santos et al. 2015). Overall, the genes compared show a high degree of conservation. One limitation of this analysis is the approximately 240 Ma of evolutionary distance between *N. vespilloides* and *T. castaneum* (Hunt et al. 2007). Moving forward, it would be interesting to see how robust these results are to other types of analyses of molecular evolution and as more beetle species over a range of phylogenetic distances are available for comparison.

Beetles are typically described as lacking DNA methylation, based on *T. castaneum* (Glastad et al. 2015; Yan et al. 2015), although the sequence for Dmnt3 has been described from transcriptomic data of a dung beetle (*Onthophagus taurus*; Choi et al. 2010) and differential methylation associated with development investigated in this beetle with amplified fragment length polymorphisms (Snell-Rood et al. 2013). In contrast to other beetles with sequenced genomes, we have direct evidence for DNA methylation of the *N. vespilloides* genome and our works show that lacking methylation is not a general feature of Coleoptera. In fact, methylation in *N. vespilloides* looks very similar to most other insects with active systems of methylation. *Nicrophorus vespilloides* has DNA methylation that is restricted to CpG sites at levels similar to honey bees (Lyko et al. 2010) and the jewel wasp *Na. vitripennis* (Wang et al. 2013), the ants *C. floridanus* and *Harpegnathos saltator* (Bonasio et al. 2012), a grasshopper *Schistocerca gregaria* (Falckenhayn et al. 2013), a locust *Locusta migratoria* (Wang et al. 2014), and the silkworm moth *Bombyx mori* (Xiang et al. 2010). Methylation is concentrated within the exons of genes as seen with honey bees (Lyko et al. 2010), ants (Bonasio et al. 2012), the jewel wasp (Wang et al. 2013), but different from a locust (Wang et al. 2014), silkworm moth (Xiang et al. 2010) and termite (Terrapon et al. 2014). Methylation was also found in the UTRs, a pattern also reported in *C. floridanus* and *H. saltator* (Bonasio et al. 2012). Methylation peaks at the beginning of the second exon, a pattern seen in ants (Bonasio et al. 2012) and the jewel wasp (Wang et al. 2013). The methylation status of genes in *N. vespilloides* appears to be evolutionarily conserved compared with jewel wasp, as true for honey bee compared with pea aphid (Hunt et al. 2010) and jewel wasp compared with honey bee (Wang et al. 2013).

It is intriguing that a social beetle, but not a nonsocial beetle, has DNA methylation. Differential DNA methylation has been implicated in the transition between behavioral states in social insects (Lyko et al. 2010; Bonasio et al. 2012; Herb et al. 2012; Terrapon et al. 2014). Because *N. vespilloides* demonstrates dramatic and reversible switches in behavioral states across a breeding cycle, and can have multiple breeding cycles, we hypothesize that DNA methylation is an epigenetic mechanism that influences these behavioral transitions.

Studies of the genetic basis and evolution of complex social behavior have focused on specific genes, with conflicting results. However, these are mostly focused on division of labor in the eusocial Hymenoptera (Zayed and Robinson 2012; Rehan and Toth 2015). The addition of the *N. vespilloides* genome allows us to expand beyond hymenopteran-specific aspects of social behavior, and allows us to begin to address broader categories of social traits. Although there are numerous aspects of the life history of burying beetles that make them unique (Eggert and Müller 1997; Scott 1998), here we have emphasized the value of using *N. vespilloides* as a model for studying family social interactions and social evolution. These beetles are particularly suited for questions of parental care because the phenotype is robust and readily measured, contains diverse subbehaviors that are reliably observed and scored, can vary between males and females in the context in which it is expressed, and is highly replicable (Walling et al*.* 2008). With the addition of the *N. vespilloides* genome, we have a taxonomically diverse arsenal of phenotypically overlapping organisms to look for phylogenetically independent genomic mechanisms and signatures of evolution, conservation, and novelty.

## Supplementary Material

Supplementary files S1 and S2, figures S1 and S2, tables S1–S7 are available at *Genome Biology and Evolution* online (http://www.gbe.oxfordjournals.org/).

Supplementary Data
