# Supplementary material for: The Genome and Methylome of a Beetle with Complex Social Behavior, Nicrophorus vespilloides (Coleoptera: Silphidae)
Source: Genome Biol Evol. 2015 Oct 9;7(12):3383–96. doi: 10.1093/gbe/evv194 (PMC4700941; doi:10.1093/gbe/evv194)
Supplement: Supplementary Data [file supp_7_12_3383__index.html]

The Genome and Methylome of a Beetle with Complex Social Behavior, Nicrophorus vespilloides (Coleoptera: Silphidae) — Supplementary Data 

# The Genome and Methylome of a Beetle with Complex Social Behavior, *Nicrophorus vespilloides* (Coleoptera: Silphidae)

## Supplementary Data

files

- Supplementary Data - xls file
- Supplementary Data - txt file
- Supplementary Data - pdf file
