# Supplementary material for: The Genome and Methylome of a Beetle with Complex Social Behavior, Nicrophorus vespilloides (Coleoptera: Silphidae)
Source: Genome Biol Evol. 2015 Oct 9;7(12):3383–96. doi: 10.1093/gbe/evv194 (PMC4700941; doi:10.1093/gbe/evv194)
Supplement: Supplementary Data [file supp_evv194_Nvespilloides_genome_paper_Cunningham_EtAl_GBE_SI_Revision.pdf]

Supplementary material

Figures.

Figure S1. Flow cytometry estimate of genome size.

Figure S2. Distribution *dN*, *dS*, and *ω* for the 25 *N. vespilloides* genes showing a differential rate of sequence evolution compared with the other two beetles.

Tables.

Table S1. Summary statistics of data used to assemble the genome (type x coverage)

Table S2. List of species that produced best hits to *N. vespilloides* gene models.

Table S3. Summary statistics for the repeat annotation.

Table S4. Summary statistics of INFERNAL annotation.

Table S5. Summary statistics for the bisulfite sequencing and mapping.

Table S6. Summary statistics for the partitioning of methylated CpG by gene model elements.

Table S7. Summary of all enriched Gene Ontology (GO) terms of the methylated genes.

Files.

File S1. Summary of expanded and contracted gene families identified by CAFÉ.

File S2. Summary of the 2,782 methylated genes.

Figures

Figure S1.

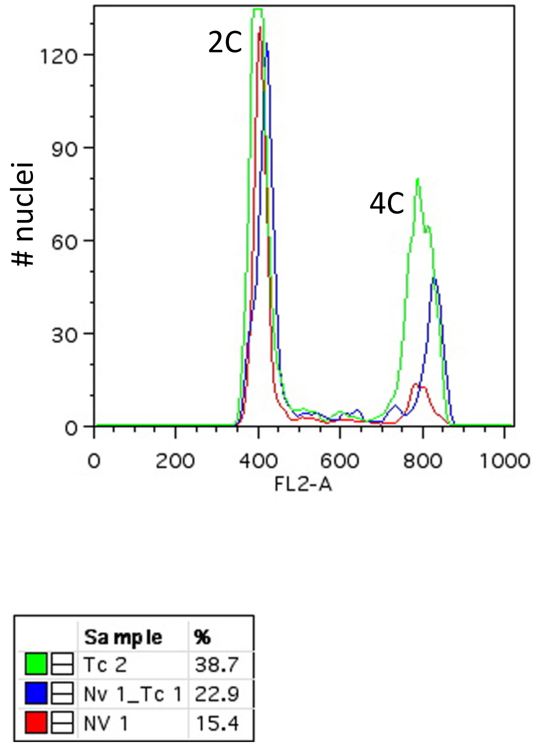

Figure S1. Histogram showing the highly overlapping florescence intensity of *Nicrophorus vespilloides* (Nv) and *Tribolium castaneum* (Tc) nuclei during fluorescent nuclear cytometry. *Tribolium castaneum*'s genome size is estimated to be 204 Mb. From this evidence we estimated the genome size of *N. vespilloides* to be of extremely similar size, nominally at 204 Mb. Fluorescent peaks for nuclei with both 2C and 4C DNA content align for both insects after staining with propidium iodide.

Figure S2.

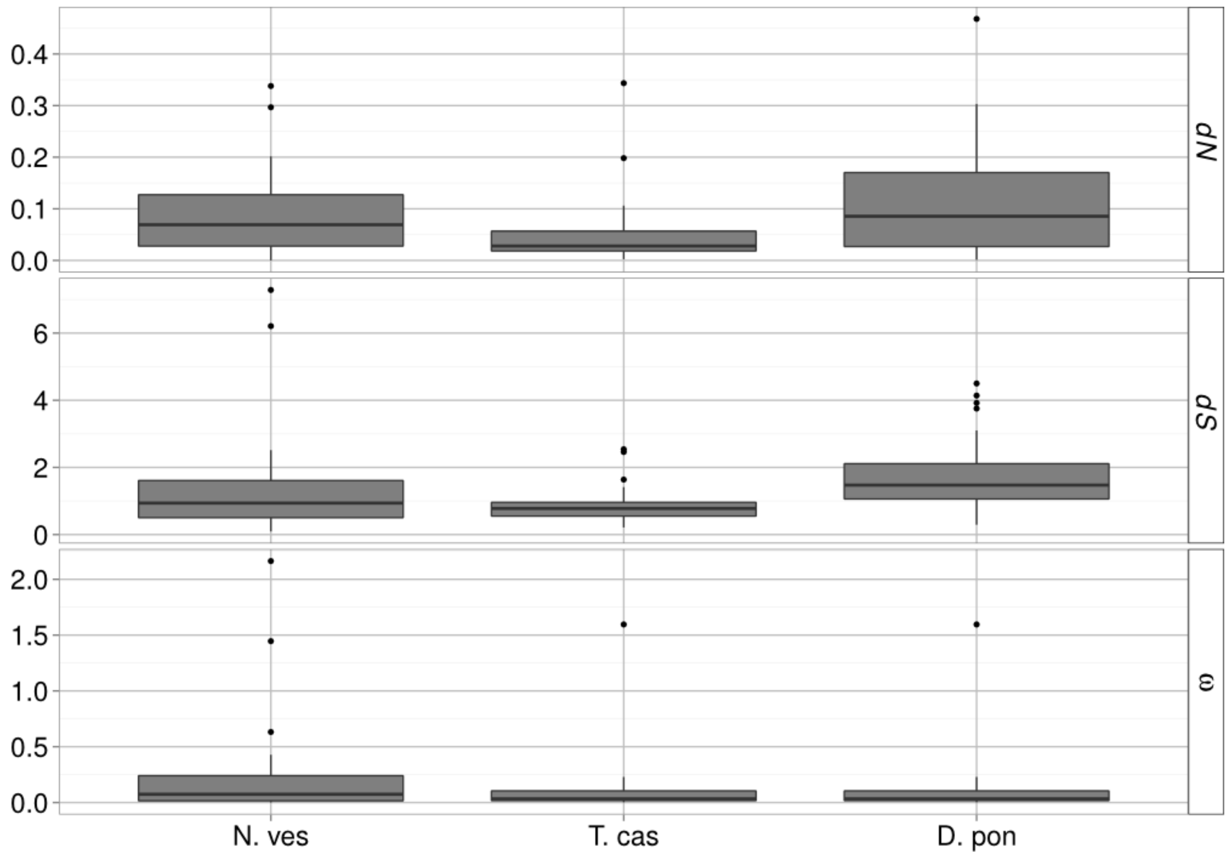

Figure S2. Distribution *dN*, *dS*, and *ω* for the 25 genes, filtered as that *dN*, *dS*, and *ω* are all < 10, showing evidence of differential rates of sequence evolution on the *Nicrophorus vespilloides* (N. ves) lineage compared with the *Tribolium castaneum* (T. cas) and *Dendroctonus ponderosae* (D. pon) lineages.

Supplementary Tables

Table S1. Summary statistics for the types of sequencing information used to assemble the *N. vespilloides* genome.

Sequencing Type		Number of Reads	Length of Reads (bp)	Estimated Coverage
<u>Illumina HiSeq Short Reads</u>				
	Paired End	180,754,627	90 - 99	175
	Singletons	22,469,671	90 - 99	11
	FLASH-combined	25,140,940	90 - 190	2
<u>Pacific Bioscience CLR's</u>				
		704,692	6,300 - 43,834	36

CLR= Continuous Long Reads

BioNano Genomics Genome Map	Number of Molecules	Length of Molecules	Estimated Coverage
<u>Irys Single Molecule Maps</u>			
	170,752	>150 kb	318

Table S2. Listing of all “best hits” against *N. vespilloides* gene models for all species that returned five or more best hits. Species are listed with their UniProtKb Organism Code and their phylogenetic Order, Class, and Phylum.

UniProtKB Organism_Code	Count_of_ Best_Hits	Taxon_Order	Taxon_Class	Taxon_Phylum
TRICA:	6969	Coleoptera	Insecta	Arthropoda
DENPD:	1368	Coleoptera	Insecta	Arthropoda
ANOGL:	743	Coleoptera	Insecta	Arthropoda
ZOONE:	383	Blattodea	Insecta	Arthropoda
APIME:	122	Hymenoptera	Insecta	Arthropoda
NASVI:	110	Hymenoptera	Insecta	Arthropoda
ACYPI:	108	Hemiptera	Insecta	Arthropoda
DANPL:	95	Lepidoptera	Insecta	Arthropoda
BOMMO:	83	Lepidoptera	Insecta	Arthropoda
9DIPT:	78	Diptera	Insecta	Arthropoda
RHOPR:	74	Hemiptera	Insecta	Arthropoda
HARSA:	67	Hymenoptera	Insecta	Arthropoda
CERBI:	66	Hymenoptera	Insecta	Arthropoda
PEDHC:	64	Phthiraptera	Insecta	Arthropoda
CAMFO:	63	Hymenoptera	Insecta	Arthropoda
AEDAE:	62	Diptera	Insecta	Arthropoda
ANOGA:	55	Diptera	Insecta	Arthropoda
CULQU:	52	Diptera	Insecta	Arthropoda
SOLIN:	49	Hymenoptera	Insecta	Arthropoda
ACREC:	40	Hymenoptera	Insecta	Arthropoda
ATTCE:	39	Hymenoptera	Insecta	Arthropoda
TENMO:	30	Coleoptera	Insecta	Arthropoda
9COLE:	30	Coleoptera	Insecta	Arthropoda
LEPDE:	29	Coleoptera	Insecta	Arthropoda
TRIIF:	29	Hemiptera	Insecta	Arthropoda
9HEMI:	28	Hemiptera	Insecta	Arthropoda
9NEOP:	28	Neoptera	Insecta	Arthropoda
DROWI:	27	Diptera	Insecta	Arthropoda
ANODA:	27	Diptera	Insecta	Arthropoda
APICE:	25	Hymenoptera	Insecta	Arthropoda
9ARAC:	24	Unknown	Arachnida	Arthropoda
AEDAL:	21	Diptera	Insecta	Arthropoda
CERCA:	19	Diptera	Insecta	Arthropoda
MUSDO:	18	Diptera	Insecta	Arthropoda
DROPS:	17	Diptera	Insecta	Arthropoda
MEGSC:	17	Diptera	Insecta	Arthropoda

DAPPU:	16	Cladocera	Branchiopoda	Arthropoda
9HYME:	15	Hymenoptera	Insecta	Arthropoda
STRMM:	15	Geophilomorpha	Linotaeniidae	Arthropoda
BACDO:	15	Diptera	Insecta	Arthropoda
DROME:	14	Diptera	Insecta	Arthropoda
DROYA:	14	Diptera	Insecta	Arthropoda
PAPPL:	13	Lepidoptera	Insecta	Arthropoda
DROGR:	13	Diptera	Insecta	Arthropoda
DROAN:	12	Diptera	Insecta	Arthropoda
9SCAR:	11	Coleoptera	Insecta	Arthropoda
9ACAR:	10	Unknown	Arachnida	Arthropoda
PAPXU:	10	Lepidoptera	Insecta	Arthropoda
DROVI:	10	Diptera	Insecta	Arthropoda
9CUCU:	9	Coleoptera	Insecta	Arthropoda
BRAFL:	8	Amphioxiformes	Leptocardii	Chordata
DROMO:	8	Diptera	Insecta	Arthropoda
ANOAQ:	8	Diptera	Insecta	Arthropoda
CHRTR:	8	Coleoptera	Insecta	Arthropoda
IXOSC:	7	Ixodidae	Arachnida	Arthropoda
DROSI:	7	Diptera	Insecta	Arthropoda
PHYDV:	7	Coleoptera	Insecta	Arthropoda
COPFO:	6	Blattodea	Insecta	Arthropoda
DROER:	6	Diptera	Insecta	Arthropoda
CTEFE:	6	Siphonaptera	Insecta	Arthropoda
BIPLU:	6	Coleoptera	Insecta	Arthropoda
PERAM:	6	Blattodea	Insecta	Arthropoda
PELSI:	5	Testudines	Sauropsida	Chordata
CAVPO:	5	Rodentia	Mammalia	Chordata
CRAGI:	5	Ostreoida	Bivalvia	Mollusca

Table S3. Output of RepeatMasker using the *de novo* repeat library generated by RepeatModleler for *N. vespilloides* reporting only classes that had identified members.

Table S3a. Repeat content of *N. vespilloides* genome ranked by prevalence.
Only classes that had identified members are reported.

Repeat Type	# of elements	Length (bp)	Percent
Unclassified	95,529	11,974,327	6.13
DNA elements	51,488	6,547,291	3.35
hAT-Charlie	8,642	1,045,262	0.54
TcMar-Tigger	28,343	3,663,375	1.88
Simple repeats	103,867	4,372,611	2.24
Low complexity	22,693	1,102,225	0.56
LTR's	3,473	744,930	0.38
LINE's	2,871	399,035	0.2
LINE1	180	85,506	0.04
LINE2	230	21,139	0.01
SINE's	516	75,259	0.04
Satellites:	85	36,130	0.02
Total		25,098,214	12.85

Table S3b. Repeat content of *T. castaneum* using the de novo repeat library of *N. vespilloides* ranked by prevalence.
Only classes that had identified members are reported.

Repeat Type	# of elements	Length (bp)	Percent
Simple repeats	38,094	1,771,969	1.17
Low complexity	10,672	524,751	0.35
LINE's	238	66,163	0.04
LINE2	5	826	0
DNA elements	495	58,649	0.04
hAT-Charlie	199	18,342	0.01
TcMar-Tigger	37	3,658	0
Unclassified:	530	58,456	0.04
SINE's	108	7,834	0.01
Satellites	5	1,024	0
LTR's	3	421	0
Total		2,488,935	1.64

Table S4. Summary of INFERNAL output classified by element and ranked by prevalence.

Infernal description	Count	Infernal description	Count	Infernal description	Count
tRNA	375	mir-11	2	let-7	1
U1	34	SNORA73	2	SNORA74	1
U3	23	MIR396	2	snoU43	1
tRNA-Sec	21	lin-4	2	ToxI	1
ACEA_U3	18	snoMe28S-Am982	2	Arthropod_7SK	1
Plant_U3	18	mir-219	2	mir-81	1
Fungi_U3	18	RNaseP_nuc	2	U11	1
snosnR61	12	mir-282	2	U12	1
U6	10	5S_rRNA	2	mir-210	1
SNORD31	9	ceN58	2	mir-944	1
mir-548	8	RNase_MRP	2	mir-580	1
mir-9	8	mir-71	2	mir-583	1
Metazoa_SRP	7	mir-208	2	mir-582	1
Protozoa_SRP	6	mir-927	2	mir-981	1
Plant_SRP	6	mir-iab-4	2	mir-190	1
Dictyostelium_SRP	6	mir-67	2	snoMe28S-Cm2645	1
Fungi_SRP	6	mir-609	2	mir-353	1
PK-G12rRNA	6	mir-276	2	mir-965	1
5_8S_rRNA	5	mir-578	2	mir-283	1
mir-2	5	SNORD36	2	mir-996	1
U4	5	mir-263	2	mir-995	1
mir-598	5	MIR1444	1	SNORD18	1
snopsi18S-1377	5	mir-395	1	mir-172	1
SSU_rRNA_bacteria	4	mir-77	1	mir-449	1
SSU_rRNA_archaea	4	SpF11_sRNA	1	mir-590	1
K_chan_RES	4	mir-317	1	MIR2118	1
mir-191	4	mir-31	1	U4atac	1
SSU_rRNA_eukarya	4	mir-36	1	MIR1023	1
snosnR60_Z15	4	SCARNA8	1	mir-277	1
mir-10	4	mir-320	1	mir-275	1
mir-279	4	mir-124	1	MIR475	1
SSU_rRNA_microsporidia	4	snoR639	1	mir-184	1
Archaea_SRP	3	mir-3	1	SNORA35	1
MIR530	3	mir-1	1	snoU18	1
MIR529	3	mir-242	1	SNORA13	1
U6atac	3	mir-7	1	mir-145	1
LSU_rRNA_bacteria	3	snoU83	1	mir-142	1
mir-46	2	mir-932	1	mir-308	1
ceN84	2	bantam	1	mir-75	1
mir-252	2	Sphinx_2	1	mir-577	1
mir-315	2	Sphinx_1	1	mir-305	1

mir-33	2	snoR442	1
mir-929	2	MIR535	1
mir-556	2	mir-14	1
mir-8	2	mir-15	1
mir-29	2	rox1	1
mir-137	2	mir-12	1
mir-133	2	SNORD70	1

Table S5. Table lists the summary statistics of each biological replicate for the methylC-sequencing. Table list the non-conversion rate of lambda DNA, read length, number of raw reads, number of mapped reads, and the estimated coverage depth across the genome.

No	Sample	Non-conversion rate	Read length (nt)	Raw reads	Mapped reads		Genome coverage
1	*N. vespilloides rep1*	0.10%	150	49,796,305	14,002,578	28.12%	10.75
2	*N. vespilloides rep2*	0.10%	150	45,783,959	13,023,930	28.45%	10.00
3	*N. vespilloides rep3*	0.10%	150	43,026,042	12,631,940	29.36%	9.70
4	*T. castaneum rep1*	0.11%	150	42,828,384	13,935,486	32.54%	9.94
5	*T. castaneum rep2*	0.11%	150	40,502,983	13,080,829	32.30%	9.33
6	*T. castaneum rep3*	0.11%	150	40,570,669	13,329,917	32.86%	9.51
7	*N. vitripennis*	1.29%	84	27,766,713	13,542,659	48.77%	3.85

Table S6. Table lists the summary statistics of each *N. vespilloides* biological replicate partitioned among gene model elements. Table lists methylated CpG among whole genome, 1kb upstream of genes, 5´ UTR's, 3´ UTR's, Exons, Introns, and Intergenic regions.

Sample	Total mC sites	Upstream		5´ UTR		3´ UTR		Exon		Intron		Intergenic	
	Number	Number	%	Number	%	Number	%	Number	%	Number	%	Number	%
N vespilloides rep1	30,759	3,325	10.81	1,604	5.74	1,832	5.96	19,251	62.59	3,133	10.19	1,614	5.25
N vespilloides rep2	27,089	2,951	10.89	1,443	5.86	1,659	6.12	16,870	62.28	2,778	10.26	1,388	5.12
N vespilloides rep3	29,826	3,163	10.60	1,531	5.65	1,768	5.93	18,731	62.80	3,109	10.42	1,524	5.11
Mean	29,225	3,146	10.77	1,526	5.75	1,753	6.00	18,284	62.55	3,007	10.29	1,509	5.16
Standard Dev.	1907	188	0.1486	81	0.1070	87	0.1063	1252	0.2638	198	0.1225	114	0.0757

Table S7. Summary of the Gene Ontology (GO) enrichment analysis of the methylated genes.

GO Term	Ontology	Description	FDR-adjusted *P* value
GO:0044260	P	cellular macromolecule metabolic process	4.00E-06
GO:0043170	P	macromolecule metabolic process	2.10E-05
GO:0044267	P	cellular protein metabolic process	0.00016
GO:0010467	P	gene expression	0.00037
GO:0043412	P	macromolecule modification	0.00044
GO:0009059	P	macromolecule biosynthetic process	0.0011
GO:0034645	P	cellular macromolecule biosynthetic process	0.0014
GO:0019538	P	protein metabolic process	0.0014
GO:0006139	P	nucleobase, nucleoside, nucleotide and nucleic acid metabolic process	0.0055
GO:0006464	P	protein modification process	0.0066
GO:0006259	P	DNA metabolic process	0.011
GO:0044237	P	cellular metabolic process	0.019
GO:0007049	P	cell cycle	0.034
GO:0006412	P	translation	0.044
GO:0003676	F	nucleic acid binding	0.0005
GO:0003723	F	RNA binding	0.0005
GO:0008135	F	translation factor activity, nucleic acid binding	0.0012
GO:0043231	C	intracellular membrane-bounded organelle	1.40E-05
GO:0043227	C	membrane-bounded organelle	1.40E-05
GO:0005634	C	nucleus	1.40E-05
GO:0044424	C	intracellular part	4.50E-05
GO:0043229	C	intracellular organelle	4.50E-05
GO:0043226	C	organelle	4.50E-05
GO:0005622	C	intracellular	8.20E-05
GO:0032991	C	macromolecular complex	0.00017
GO:0044428	C	nuclear part	0.00083
GO:0044422	C	organelle part	0.00083
GO:0044446	C	intracellular organelle part	0.00083
GO:0043234	C	protein complex	0.0016
GO:0043233	C	organelle lumen	0.0018
GO:0070013	C	intracellular organelle lumen	0.0018
GO:0031974	C	membrane-enclosed lumen	0.0025
GO:0030529	C	ribonucleoprotein complex	0.015
GO:0044444	C	cytoplasmic part	0.015
GO:0031981	C	nuclear lumen	0.015
GO:0005840	C	ribosome	0.015
GO:0005737	C	cytoplasm	0.015
GO:0012505	C	endomembrane system	0.02
GO:0005654	C	nucleoplasm	0.021
GO:0044464	C	cell part	0.036
GO:0005623	C	cell	0.036
